# Nontraumatic Tibial Polyethylene Insert Cone Fracture in Rotating-Platform Total Knee Arthroplasty

**DOI:** 10.1016/j.artd.2021.02.013

**Published:** 2021-04-08

**Authors:** Cillian J. Keogh, David Keohane, James A. Harty, David Mulcahy

**Affiliations:** a Department of Trauma & Orthopaedic Surgery, Cork University Hospital and South Infirmary Victoria University Hospital, Wilton, Cork, Ireland; b Department of Orthopaedic Surgery, Bon Secours Hospital, College Road, Cork, Ireland

**Keywords:** Total, Knee, Arthroplasty, Polyethylene, Fracture, Wear

## Abstract

We report a case of a fracture through the polyethylene (PE) insert cone in a rotating-platform (RP) primary total knee arthroplasty (TKA) implant. This is the first reported case of cone fracture with this particular implant. This case highlights a 65-year-old female presenting with a 4-month history of knee pain and symptoms of instability 10 years after primary RP TKA. At the time of revision surgery, the PE insert cone was found to be fractured 10 mm from the inferior surface of the PE liner. Analysis suggests that the cone fracture occurred by fatigue failure which caused loosening of the tibial tray. Clinicians should be aware that this is a potential cause of failure with an RP TKA in the setting of symptoms including instability and pain.

## Introduction

Ultra-high-molecular-weight polyethylene (UHMWPE) is a plastic or semicrystalline polymer that has been used in joint arthroplasty since 1962. The wear and failure of polyethylene (PE) liners in total knee arthroplasty (TKA) is a major limitation of its long-term success.

LCS Complete Knee system (DePuy Inc., Warsaw, IN) has an excellent track record with a documented 20-year survival rate of 97.7% [1]. It was designed to restore anatomical function and motion and to overcome the damaging effects of component wear and loosening. The rotating-platform (RP) design provides both congruity and mobility in the tibial and femoral bearing surfaces. This allows low contact stresses within the PE insert and theoretically reduces wear and minimizes loosening. LCS Complete PE inserts are manufactured from the UHMWPE GUR 1020 polymer (Celanese GmbH, Frankfurt, Germany). It is gamma-irradiated and foil-packaged in an inert environment for sterilization. In 2011, DePuy introduced AOX (DePuy Inc., Warsaw, IN) an antioxidant version of the PE insert in an attempt to improve mechanical integrity and oxidative stability. The COVERNOX (Depuy Inc., Warsaw, IN) antioxidant combines with postirradiation free radicals to stabilize them.

Fracture of the PE tibial post in fixed bearing posterior stabilized (PS) TKA is rare but well documented [[2], [3], [4]]. The post is a prominent area of PE and can be a focal point for stress within the knee. Malalignment of components has been previously hypothesized to be a risk factor for PE post fracture [5]. During extension, there is impingement anteriorly on the tibial post, while in flexion, impingement occurs posteriorly. This can lead to wear, and in a poorly balanced TKA, this wear can be severe enough to result in failure [6]. Excessive rotation between the tibial and femoral components can also cause impingement of the tibial post in the femoral cam leading to fracture [6].

In an RP TKA design, there is a distal PE insert cone that articulates with the tibial component providing a means for rotation and bearing stability. There have been 2 documented cases of tibial PE insert cone fracture in RP TKA. These were however from PS RP TKA design with a cam and post mechanism [7,8].

We report a case of a nontraumatic primary TKA failure secondary to a PE insert cone fracture in an LCS Complete (DePuy Inc., Warsaw, IN) RP TKA design. To our knowledge, this is the first reported case of cone fracture with this particular implant. Unfortunately, the patient discussed in this case has since passed away. We obtained consent from her next of kin for publication of this case report.

## Case history

**Figure 1:**
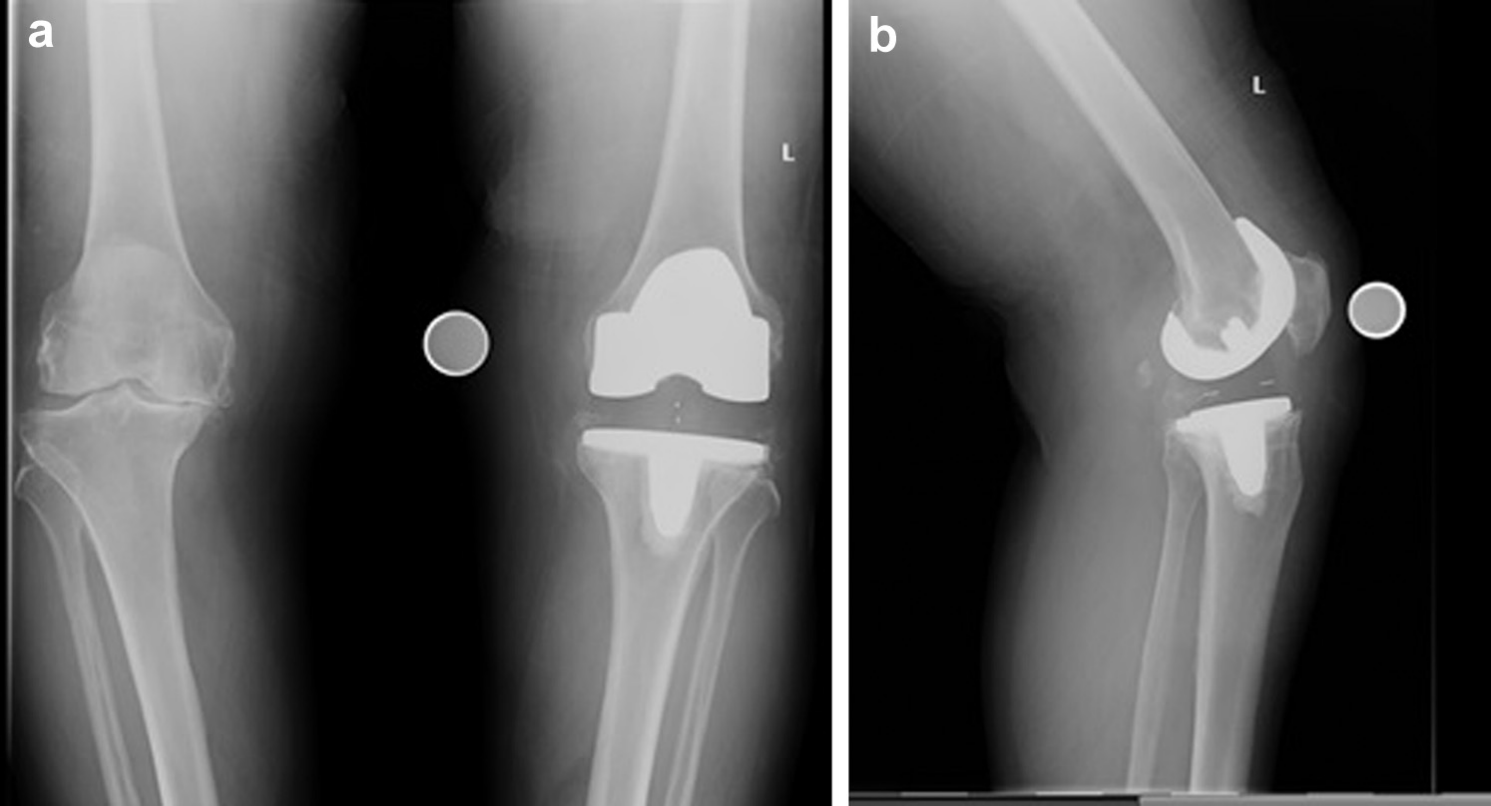
(a) Anteroposterior and (b) lateral radiographs of left TKA at presentation 10 years after index surgery showing loosening on the lateral aspect of the tibial tray.

In January 2015, 10 years and 4 months after the index procedure of primary RP left TKA, a 64-year-old lady re-presented to her primary orthopedic surgeon complaining of new symptoms of pain and a feeling of giving way in the left knee which progressed over a period of 4 months. Plain film radiographs of her left knee suggested loosening on the lateral aspect of the tibial tray with satisfactory alignment and no evidence of spinout (Fig. 1a and 1b). Serum laboratory markers for infection were all within normal limits. This was further investigated with an isotope bone scan performed in May 2015 using technetium 99m-methyl diphosphonate. This showed increased radiotracer uptake under the medial and lateral aspects of the tibial plate which confirmed loosening.

She initially presented for her index procedure in 2004 complaining of severe pain and stiffness in her left knee. On examination, she had a varus deformity of 20° with a range of motion from 10° to 100°. Her background history was significant for hypertension, peptic ulcer disease, and an elevated BMI of 36 (96 kg weight and 1.62 m height). Radiographs at the time confirmed severe tricompartmental osteoarthritis with varus deformity and lateral subluxation.

After all conservative management options were exhausted, she underwent an uncomplicated left-sided RP TKA on October 6, 2004. Using an LCS Complete RP TKA (DePuy Inc., Warsaw, IN) design and a measured resection technique, a standard plus femoral component and size 3 tibial component were cemented in place with Palacos (Heraeus Medical GmbH, Wehrheim, Germany) bone cement. Owing to the severe deformity, larger cuts were made from both the distal femur and proximal tibia. This bone resection led to a relatively thick PE RP liner of 20 mm. Her postoperative course at 6 weeks, 6 months, and 1 year was unremarkable. At the 6-week follow-up appointment, she mobilized independently, denied any pain, and had a range of motion from 0° to 120°.

**Figure 2:**
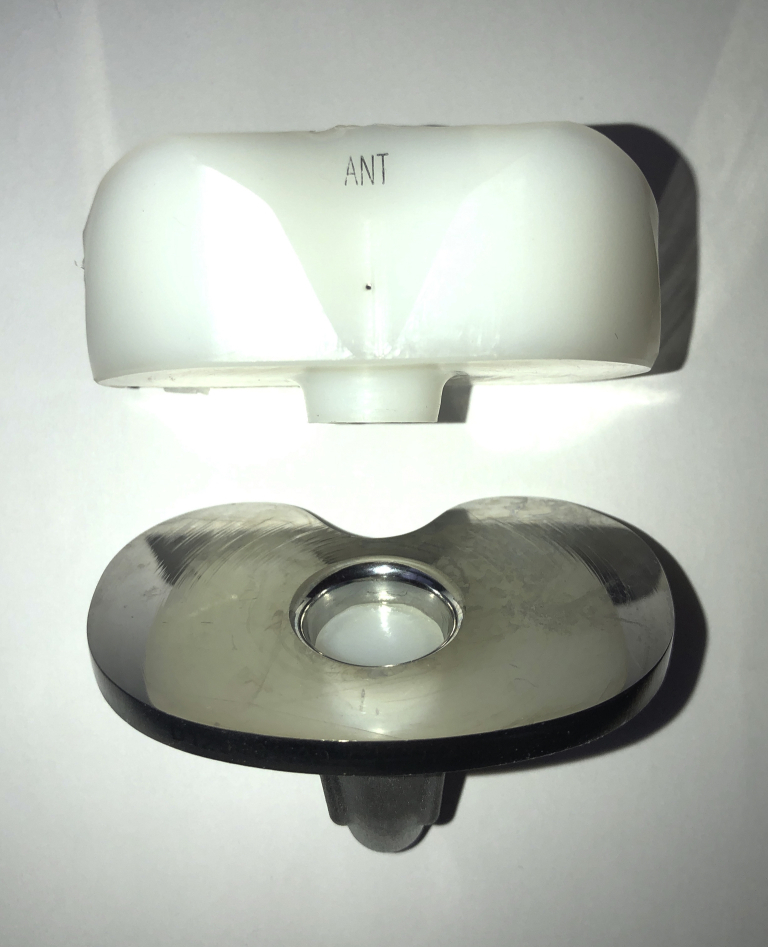
Fracture of the polyethylene insert cone 10 mm from the inferior surface of the polyethylene liner.

**Figure 3:**
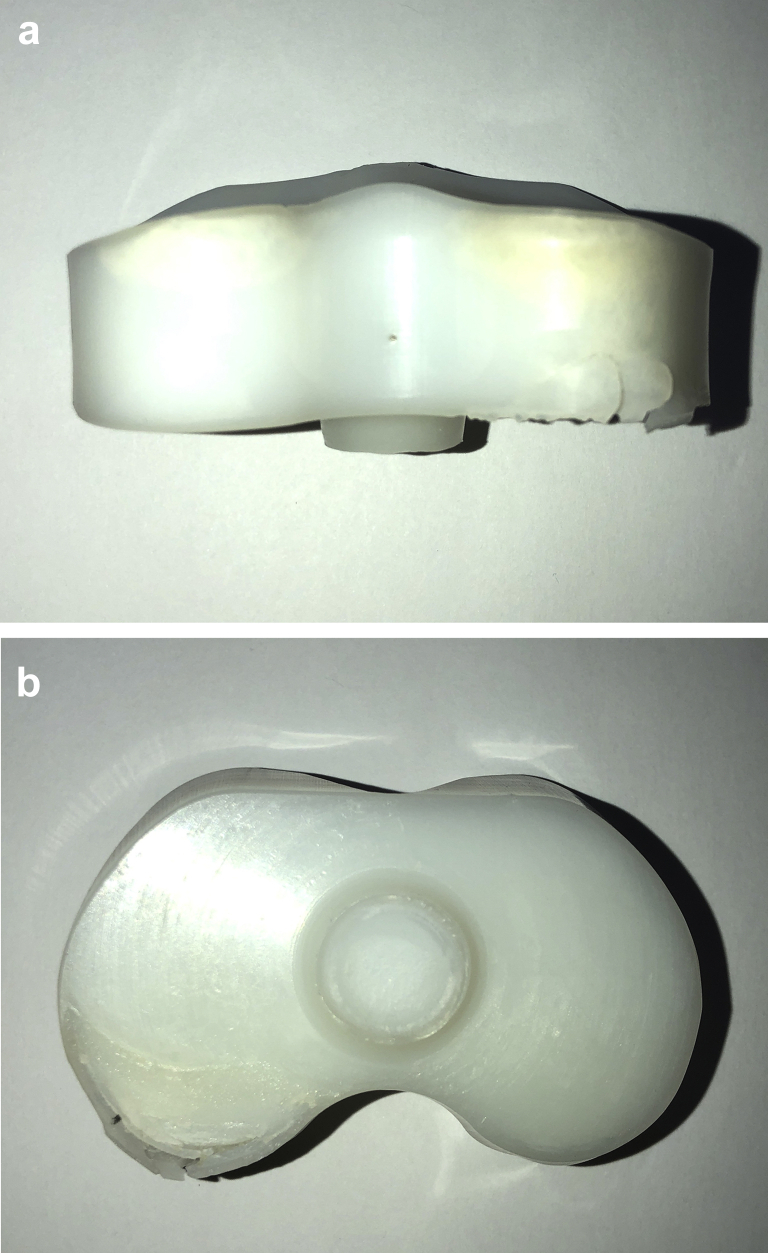
(a) Posterior view and (b) inferior view of PE insert showing a moderate amount of delaminated wear on the posteromedial aspect of the inferior surface.

**Figure 4:**
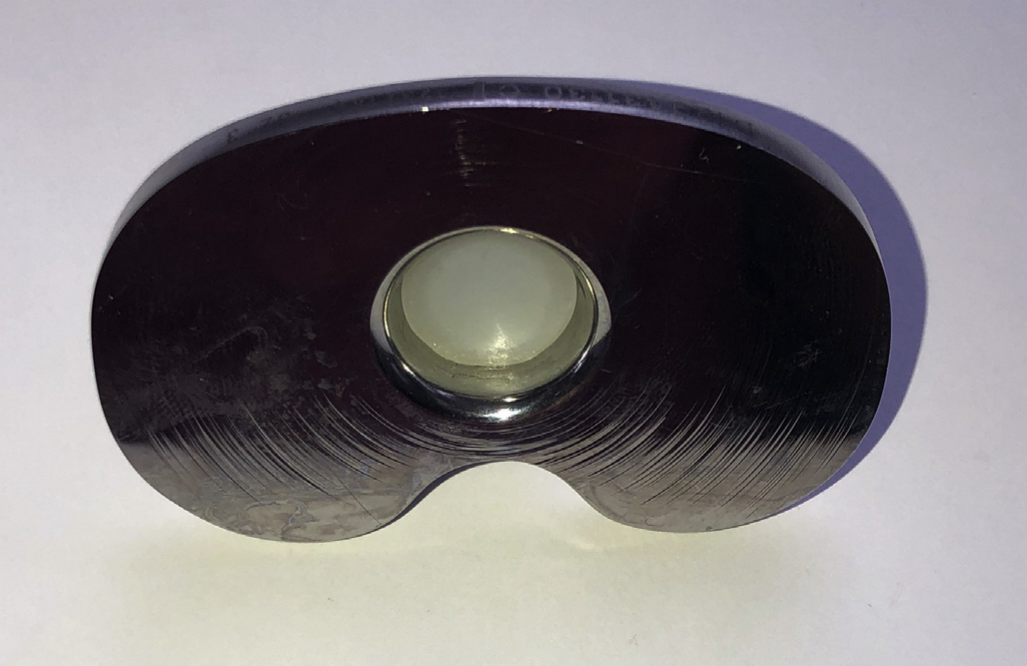
Scratches on the tibial tray surface forming concentric rings.

**Figure 5:**
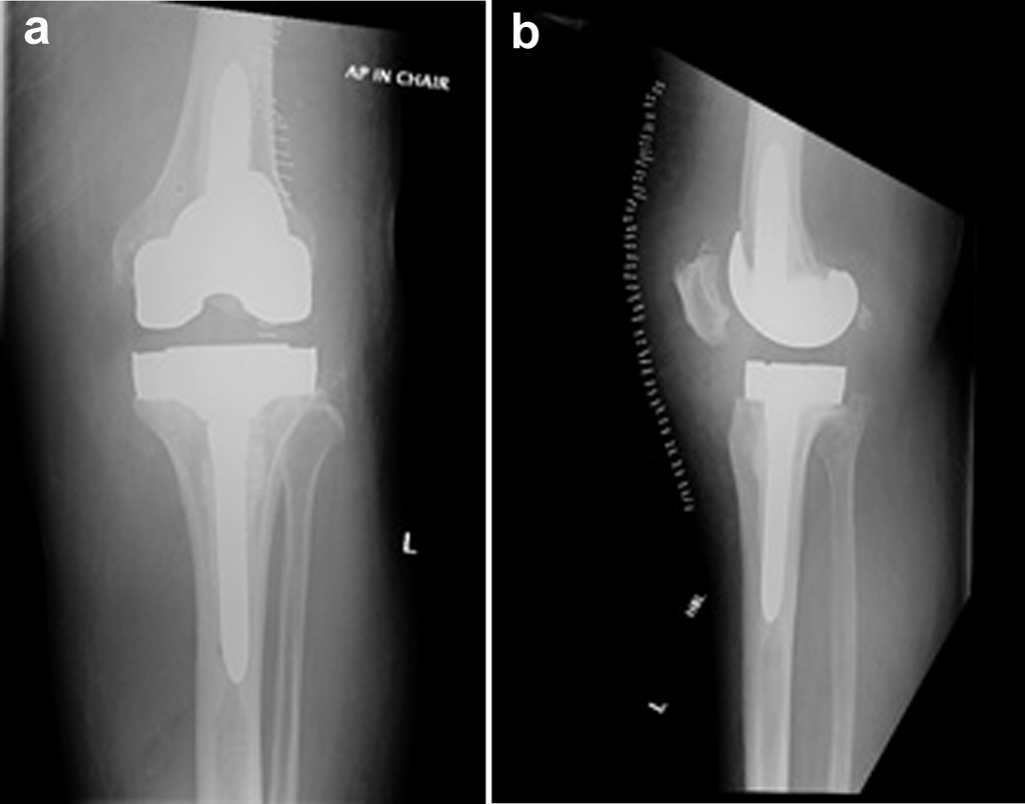
(a) Anteroposterior and (b) lateral radiographs immediately after revision TKA.

On October 7, 2015, the patient underwent revision left TKA for symptomatic aseptic loosening. At the time of revision, the PE insert cone was found to have fractured 10 mm from the inferior surface of the PE liner (Fig. 2). A complete separation of the cone from the insert had occurred such that the cone itself remained lodged within the tibial tray. The tibial component was loose, and there was metal staining (metallosis) in the soft tissues. There was a moderate amount of delaminated wear on the posteromedial aspect of the inferior surface of the PE insert (Fig. 3a and 3b). Scratches were also noted on the tibial tray surface forming concentric rings aligned with rotational movement around the center of the tray (Fig. 4). A single-stage revision was performed using a stemmed cemented Triathlon (Stryker, Kalamazoo, MI) Total Stabilizer revision knee system with 10 mm tibial augments, 10 mm distal femoral augments, and 5 mm posterior femoral augments (Fig. 5a and 5b). She complained of some mild discomfort after revision but mobilized independently with a range of motion from 0° to 90°.

An examination of the retrieved LCS implant was conducted by Dr. Harry Hothi, an orthopedic engineer, at the London Implant Retrieval Center, based at the Royal National Orthopedic Hospital, Stanmore, UK. The retrieval analysis methods used in this study were (1) macroscopic imaging and inspection using a Canon (Canon Inc., Tokyo, Japan) Mark II DSLR and Canon (Canon Inc., Tokyo, Japan) 100-mm L lens, (2) microscopy using a Keyence VHX-700F (Keyence Co., Osaka, Japan) light microscope, and (3) scanning electron microscopy (SEM; Carl Zeiss (Zeiss AG, Oberkochen, Germany) EVO 25) after gold coating of the surface features of interest.

**Figure 6:**
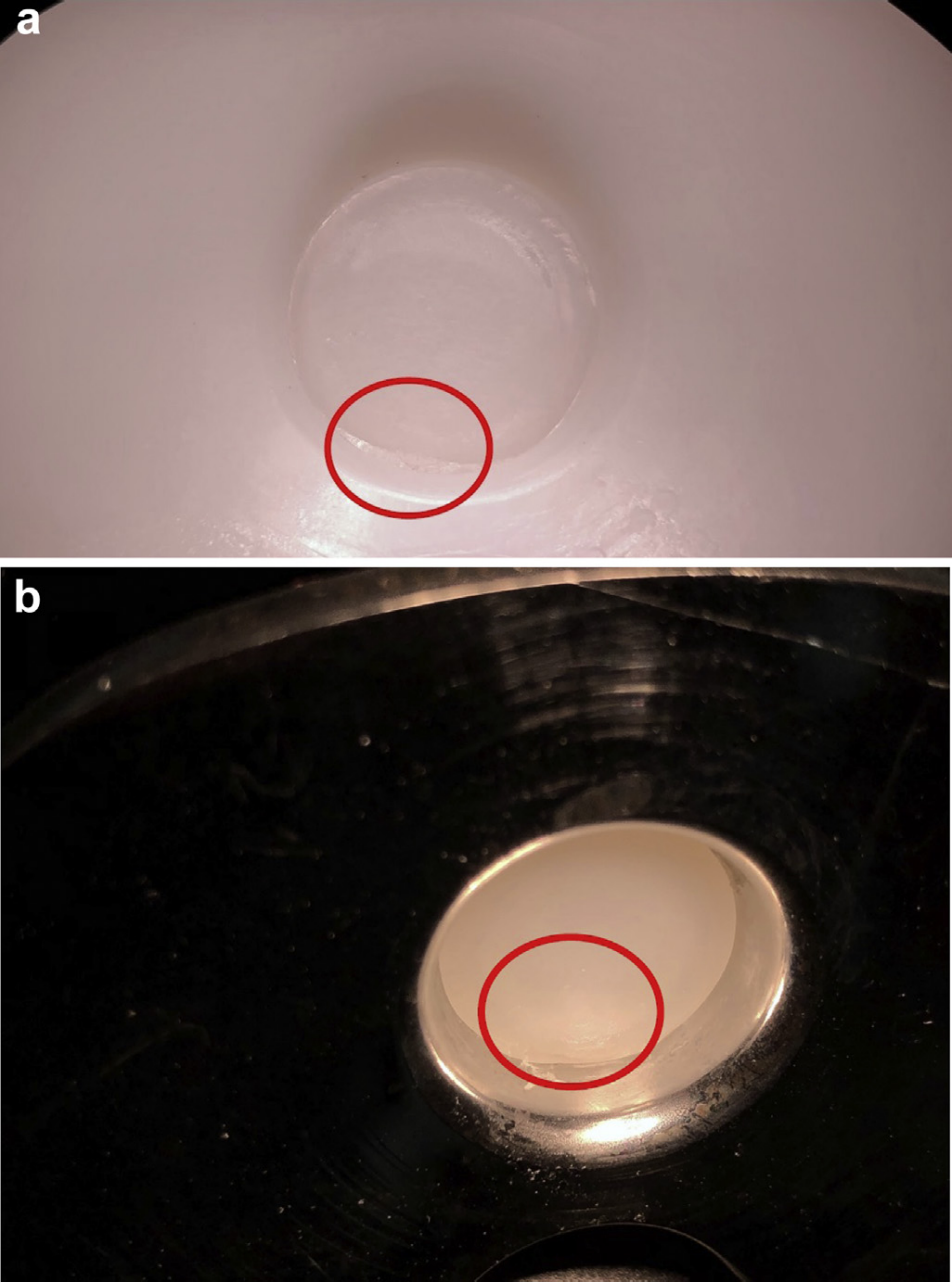
Macroscopic imaging revealed a region of great deformation at the edge of the fracture site (red circle). This was seen on the inferior aspect of the PE liner (a) and on the cone insert fractured in the tibial tray (b).

**Figure 7:**
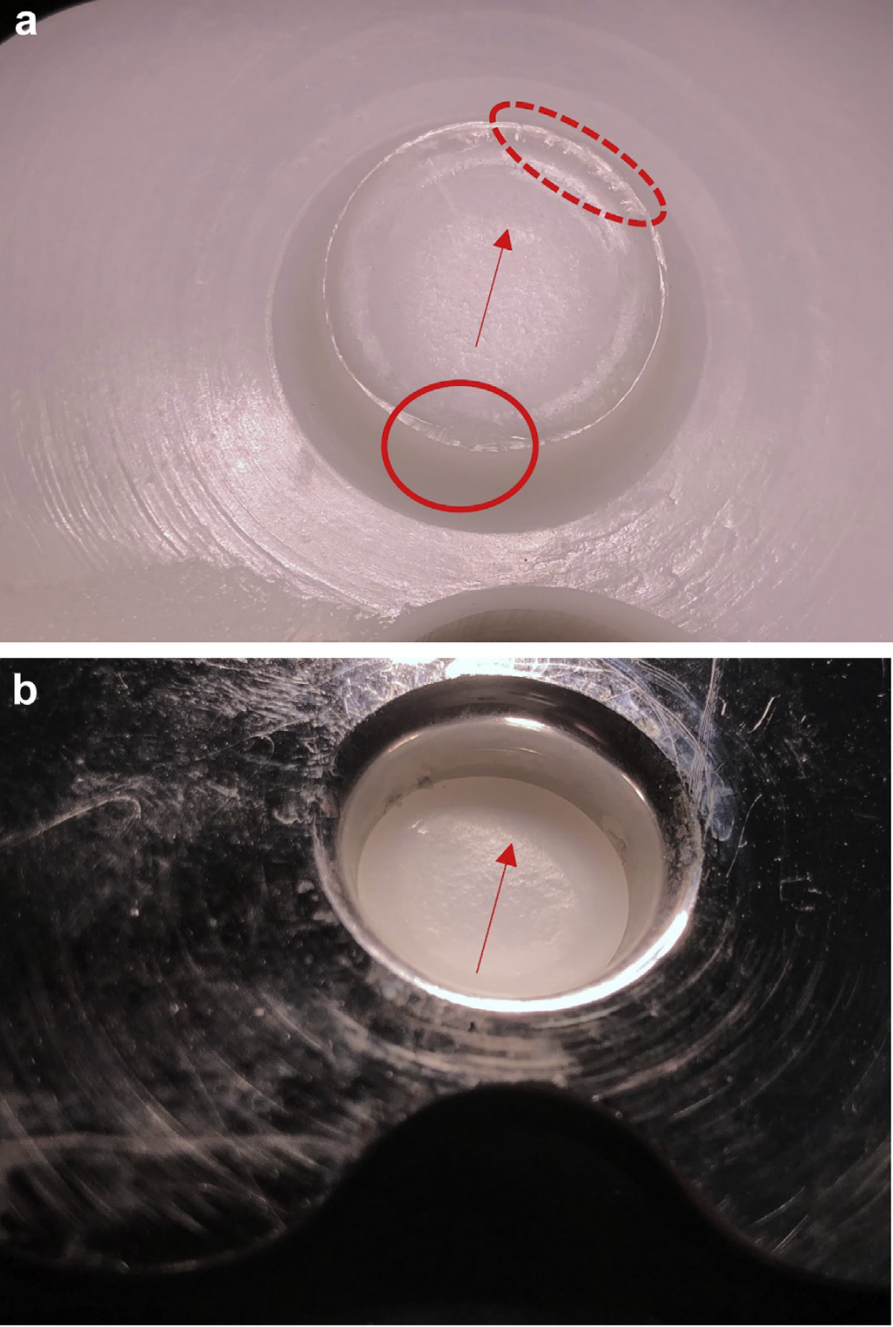
The red arrow signifies the direction of the fracture propagation with final separation in the region indicated by the dashed ellipse. The arrow head indicated the region at which the speed of fracture progression slowed down. This was seen on the inferior aspect of the PE liner (a) and on the cone insert fractured in the tibial tray (b).

Visual inspection and macroscopic imaging revealed evidence of a deformation on the cone at the edge of the fracture site on the posterior side of the insert (Fig. 6a). A corresponding change of shape was observed on the portion of the cone still lodged within the tray (Fig. 6b). This region is believed to be the fracture initiation site. A variability in the surface of the fracture site was macroscopically evident with a rougher topography emanating from the fracture initiation site and transitioning into a visibly smoother region and then a slightly raised lip on the anterior side, at the suspected point of separation (Fig. 7a and 7b).

**Figure 8:**
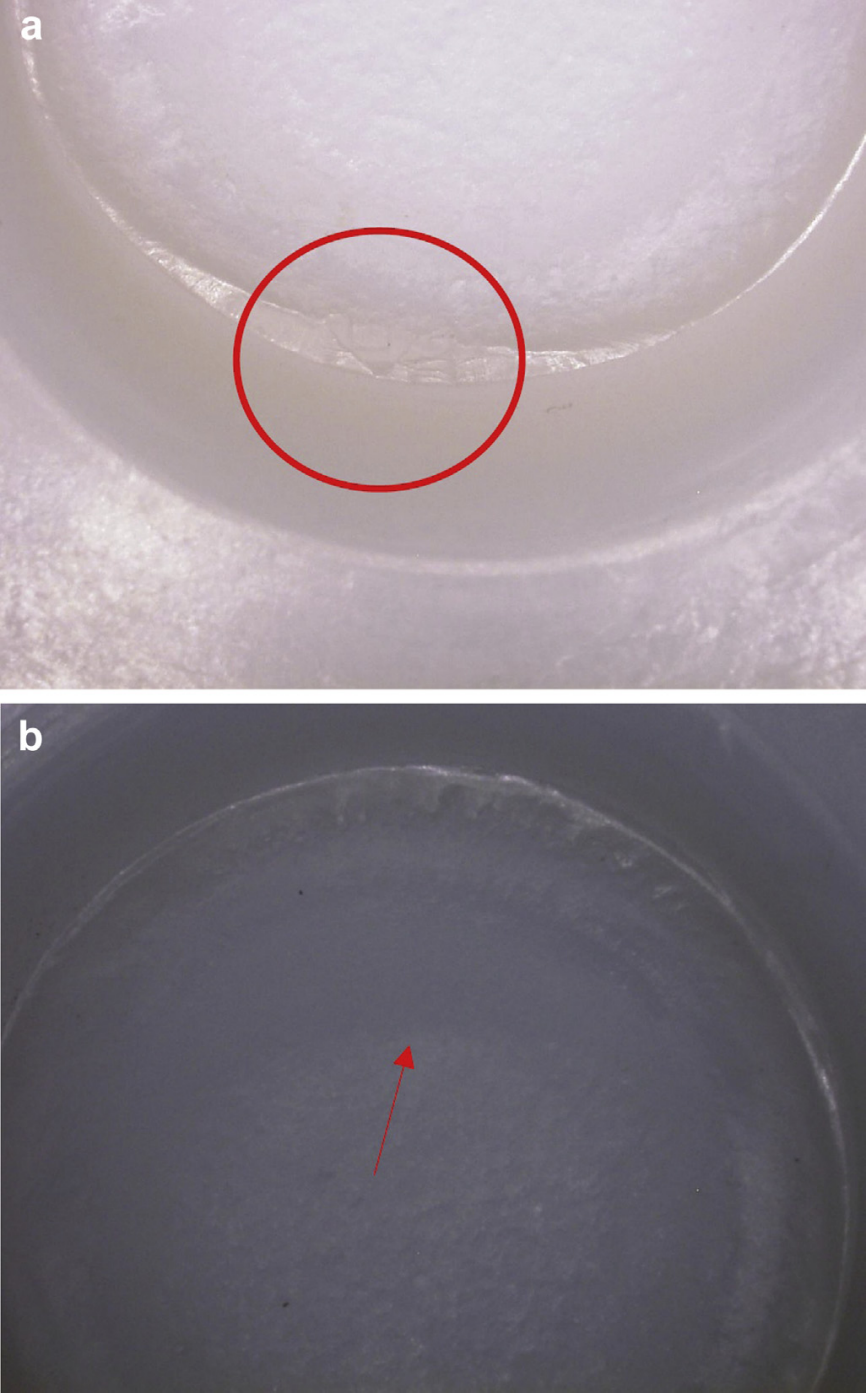
Microscopic imaging confirmed the presence of deformation at the fracture initiation site (red circle) (a) and a transition in the fracture mode (red arrow) (b).

**Figure 9:**
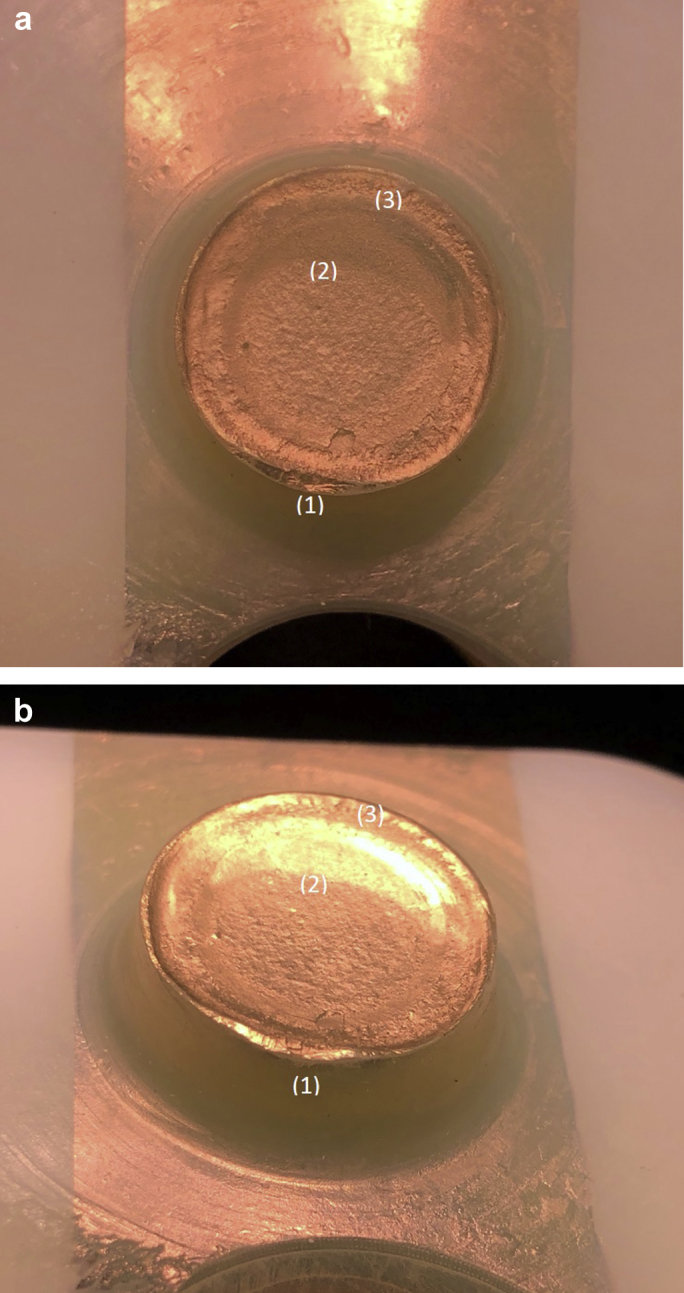
Macroscopic imaging of the gold coated surfaces of the inferior aspect of the PE liner at (a) and (b) showed clearly the (1) fracture initiation point, (2) the transition point of the mode of fracture from a rapid progression to a slower fatigue fracture and (3) the point of final separation of the cone due to ductile fracture, characterized by a raised lip in this side anteriorly. There were some indicators of beach marks following the transition at (2) however these were not at all prominent.

**Figure 10:**
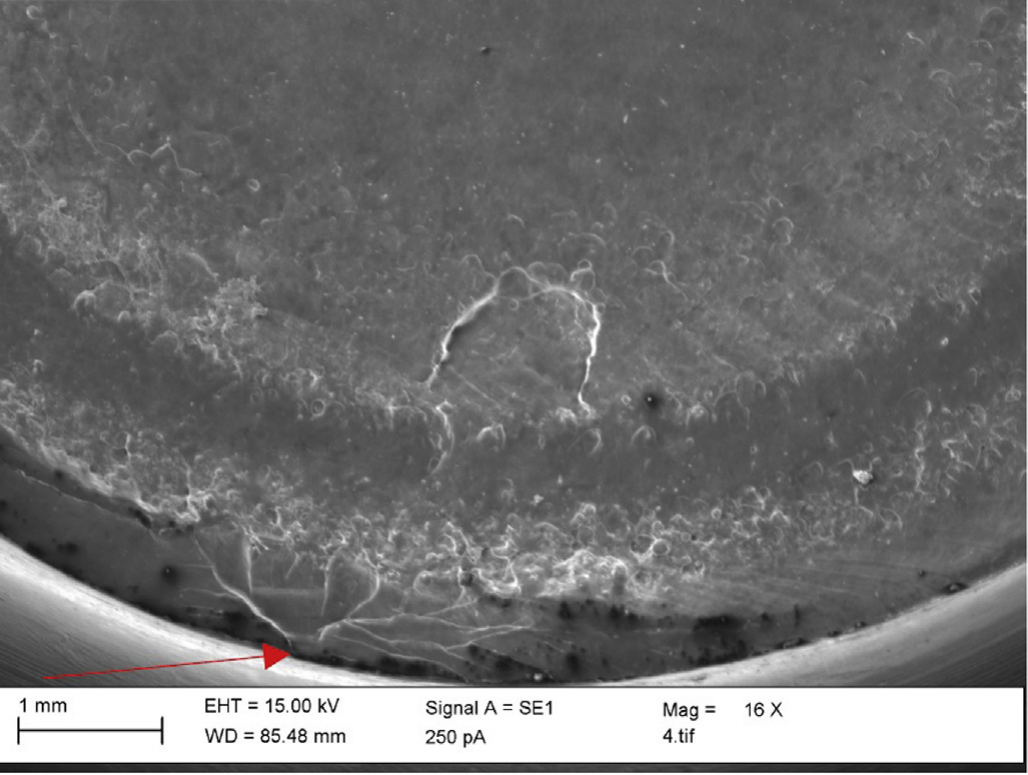
SEM imaging confirmed a clear difference in the surface damage patterns at the fracture initiation site (red arrow). This high magnification imaging did not reveal clear beach marks that are characteristic of fatigue fracture.

Microscopic imaging confirmed clear pronounced surface damage localized at the suspected fracture initiation site (Fig. 8a) and a transition in the fracture mode (Fig. 8b). In order to perform SEM analysis, the surfaces under investigation were first gold coated to allow a clearer visualization of the surface details. The component was reimaged macroscopically with the coating applied, and some evidence of beach marks characteristic of fatigue fractures were evident (Fig. 9a and 9b). It is of note however that these beach marks were not prominent and not highly prevalent, as may be normally expected in cases where a fatigue fracture has occurred. Indeed, the rougher surface topography is more characteristic of a rapid brittle fracture. SEM imaging also confirmed a clear difference in the surface damage patterns at the fracture initiation site with some slight but not very prominent beach marks (Fig. 10).

**Figure 11:**
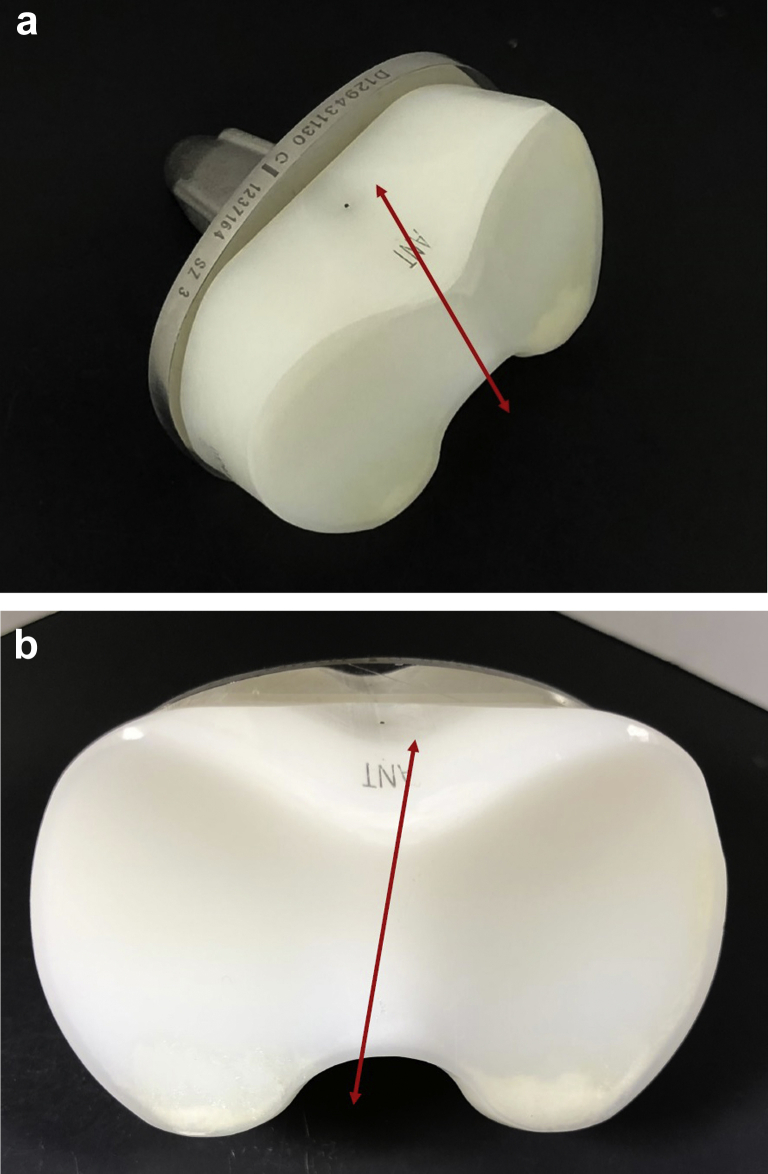
The arrows indicate the suspected direction of toggling of the insert within the tray seen in (a) and (b), ultimately leading to the fracture of the cone.

The mechanism of fracture of the cone appears to have been due to repeated rocking or cantilever stresses toward the direction of the red arrow (Fig. 11a and 11b), causing a primary fracture at the initiation site. We then observed initial rapid progression of the fracture across the cone surface followed by a slower fatigue fracture progression and finally separation of the cone due to a ductile fracture.

## Discussion

Our analysis showed that increased rocking forces which were exacerbated by a larger PE liner thickness, resection of the posterior cruciate ligament (PCL), and an elevated BMI led to the fracture of the cone portion of the PE liner. Postirradiation oxidative aging may also have embrittled the UHMWPE liner which further increased its susceptibility to fracture. It is likely that the fracture of the cone occurred first, and this initial fracture of the cone caused decreased stability and multidirectional movement of the PE liner on the broken cone and tibial tray. These factors allowed generation of asperities, wear debris, and metallosis. This subsequently resulted in induction of the inflammatory response causing osteolysis. Finally, a combination of mechanical instability and osteolysis caused loosening of the tibial tray. This catastrophic failure of the implant resulted in the patient’s symptoms of pain and instability over a 4-month period.

Several factors contribute to wear and fracture of PE in TKA. These can be divided into patient, technique, or implant-associated factors. Patient factors include activity level, age, and BMI. Surgical technique must focus on reducing wear by preserving the level of the joint line, balancing the knee in both flexion and extension, and restoring the mechanical axis of the joint with correct implant alignment. Implant factors that contribute to PE wear mainly revolve around fixed and mobile bearing implants and the way the PE is manufactured.

Fatigue failure or fracture of materials is caused by the nucleation and propagation of cracks under cyclic loading. It can be defined as a progressive and localized process which occurs because of conditions that cause fluctuating stresses and strains in one or more location and can result in the formation of cracks and the complete rupture of the component after a sufficient number of cycles [9].

Our analysis suggested that the fracture of the cone occurred because of the repeated rocking moment of the tibial PE insert. The rocking moment of a PE insert is the force times the distance of the vector to the center of the tibial component as viewed on the sagittal plane. The calculation of rocking moment is shown using the illustration by Soudry et al. in Appendix 1. The moment would therefore be increased if the body weight was increased (patient had high BMI, increasing R) and if the height or thickness of the PE insert was increased (20 mm insert used, increasing a). The increased rocking moment would increase the stress on the PE cone and would potentially decrease its S-N fatigue lifetime.

When the PCL is resected, the rocking moment produces anterior tilting of the PE liner on the tibial component in flexion [10]. This is due to posterior translation of the tibia relative to femur after resection of the PCL and therefore an anterior contact area on the PE liner [10]. Studies have shown that when the PCL is resected, the rocking moments of the PE liner are 4.3 times and 1.6 times greater at 45° and 90° of flexion, respectively, than those with PCL retention [10]. This is due to the greater distances of the contact point from the center. We posit that as our case shows that the fracture initiation site is on the posterior aspect of the PE cone and that the rocking motion is most likely anterior with flexion of the knee, the initial crack on PE cone would have formed under tension.

Sterilizing PE by gamma irradiation leads to scission of the long molecular chains and production of free radicals within the polymer matrix. The introduction of newer techniques such as gamma-inert sterilization, addition of vitamin E and antioxidants, and annealing have greatly reduced the potential for oxidation during shelf storage. However, the free radicals created still remain, and in vivo oxidation is still possible after implantation of the components in TKA [11]. Diffusion of oxygen from bodily fluids into the polymer matrix and diffusion of free radicals out of the polymer can lead to the development of a region of high subsurface oxidation and a consequent subsurface oxidized band. These postirradiation oxidative changes embrittle UHMWPE and decreases its ultimate tensile stress, toughness [12], fatigue crack propagation resistance [13], and S-N fatigue lifetime [14]. The embrittled UHMWPE may help explain the initial rapid progression of the fracture across the cone surface observed in this case.

While the 2 other documented cases of cone fracture in the literature were PS RP TKA designs, they shared the same findings of rocking stresses and in vivo oxidation of the PE liner.

## Summary

In conclusion, it is difficult to diagnose failure of the PE insert cone in a RP TKA design. While the imaging revealed loosening of the tibial tray and the patient’s symptoms warranted revision TKA, catastrophic failure of the PE insert cone was only noticeable during the revision procedure. This is the third report of a PE cone fracture in primary TKA and, to our knowledge, the first report related to this particular implant. This introduces another potential area for implant failure which clinicians should consider when patients complain of symptoms of pain and instability.

## Conflicts of interest

The authors declare that they have no known competing financial interests or personal relationships that could have appeared to influence the work reported in this article.

## Figures and Tables

**Figure S1:**
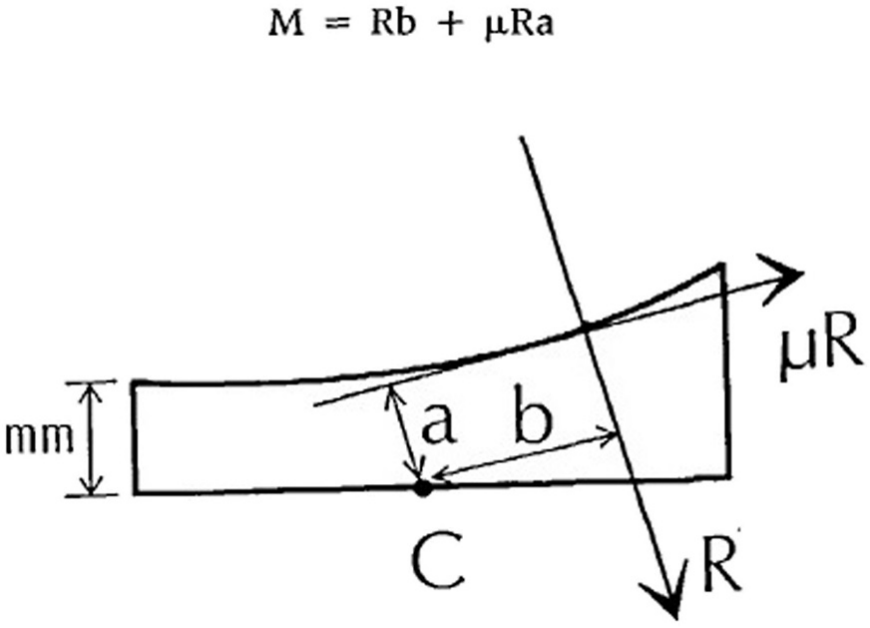


## Appendix

Appendix 1: Calculation of the rocking moment (M) which is the sum of the moments around the centre (C). The forces acting at the surface of the polyethylene liner are R and μR. a and b are the distances of the vector to the centre of the tibial component.

## Appendix A Supplementary data

Conflict of Interest Statement for Keohane

Conflict of Interest Statement for Mulcahy

Conflict of Interest Statement for Harty

Conflict of Interest Statement for Keogh
