# Supplementary material for: Nontraumatic Tibial Polyethylene Insert Cone Fracture in Rotating-Platform Total Knee Arthroplasty
Source: Arthroplast Today. 2021 Apr 8;8:283–288.e1. doi: 10.1016/j.artd.2021.02.013 (PMC8167323; doi:10.1016/j.artd.2021.02.013)
Supplement: Conflict of Interest Statement for Keogh [file mmc4.pdf]

INDIVIDUAL CONFLICT OF INTEREST STATEMENT

American Association of Hip and Knee Surgeons

(Adopted from the American Academy of Orthopaedic Surgeons disclosure statement)

The following form **must be filled out completely and submitted by each author (example, 6 authors, 6 forms). All items require a response. If there is no relevant disclosure for a given item, enter "*None.*"**

Manuscript Title: A Report of 8 cases of Polyethylene Spinout in the ATTUNE Cruciate-Retaining Rotating-Platform Total Knee Arthroplasty

1. Royalties from a company or supplier (The following conflicts were disclosed)

   NONE

2. Speakers bureau/paid presentations for a company or supplier (The following conflicts were disclosed)

   NONE

3A. Paid employee for a company or supplier (The following conflicts were disclosed)

   NONE

3B. Paid consultant for a company or supplier (The following conflicts were disclosed)

   NONE

3C. Unpaid consultants for a company or supplier (The following conflicts were disclosed)

   NONE

4. Stock or stock options in a company or supplier (The following conflicts were disclosed)

   NONE

5. Research support from a company or supplier as a Principal Investigator (The following conflicts were disclosed)

   NONE

6. Other financial or material support from a company or supplier (The following conflicts were disclosed)

   NONE

7. Royalties, financial or material support from publishers (The following conflicts were disclosed)

   NONE

8. Medical/Orthopaedic publications editorial/governing board (The following conflicts were disclosed)

   NONE

9. Board member/committee appointments for a society (The following conflicts were disclosed)

   NONE

Each author must sign AND print or type his/her name, date and submit a separate form

In addition, one BLINDED Conflict of Interest form (no author names used) should be submitted per manuscript with all author disclosures.

Mr Cillian J Keogh — [signature] — 02/12/2020

Author Name (Print or Type) — Author Signature — Date
